# Two tripartite classification systems of CD86^+^ and CD206^+^ macrophages are significantly associated with tumor recurrence in stage II-III colorectal cancer

**DOI:** 10.3389/fimmu.2023.1136875

**Published:** 2023-06-05

**Authors:** Guozeng Xu, Yuzhen Mo, Jing Li, Qingqing Wei, Fuxiang Zhou, Jian Chen

**Affiliations:** ^1^ Department of Radiation and Medical Oncology, Zhongnan Hospital of Wuhan University, Hubei, China; ^2^ Department of Oncology, Liuzhou People’s Hospital of Guangxi Medical University, Guangxi, China; ^3^ Department of Radiation Oncology, Guangzhou Red Cross Hospital of Jinan University, Guangdong, China; ^4^ Department of Medical Oncology, Yantai Yuhuangding Hospital of Qingdao University, Shandong, China

**Keywords:** CD86, CD206, colorectal cancer, macrophage polarization, tripartite classification system

## Abstract

**Introduction:**

The prognostic value of tumor-associated macrophages remains unclear in colorectal cancer (CRC). Two tripartite classification systems, namely, ratio and quantity subgroups, were investigated as the prognostic stratification tools for stage II-III CRC.

**Methods:**

We assessed the infiltration intensity of CD86^+^ and CD206^+^ macrophages in 449 cases with stage II-III disease by immunohistochemical staining. Ratio subgroups were defined by the lower- and upper-quartile points of CD206^+^/(CD86^+^+CD206^+^) macrophage ratio, including the low-, moderate-, and high-ratio subgroups. Quantity subgroups were defined by the median points of CD86^+^ and CD206^+^ macrophages and included the low-, moderate-, and high-risk subgroups. The main analysis was recurrence-free survival (RFS) and overall survival (OS).

**Results:**

Ratio subgroups (RFS/OS: HR=2.677/2.708, all *p*<0.001) and quantity subgroups (RFS/OS: HR=3.137/3.250, all *p*<0.001) could serve as independent prognostic indicators that effectively predicted survival outcomes. More importantly, log-rank test revealed that patients in the high-ratio (RFS/OS: HR=2.950/3.151, all *p*<0.001) or high-risk (RFS/OS: HR=3.453/3.711, all *p*<0.001) subgroup exhibited decreased survival outcomes after adjuvant chemotherapy. The predictive accuracy of the quantity subgroups within 48 months was higher than that of the ratio subgroups and tumor stage (all *p*<0.05).

**Conclusions:**

Ratio and quantity subgroups could serve as independent prognostic indicators that could potentially be incorporated into the tumor staging algorithm to improve prognostic stratification and provide better predictions of survival outcomes in stage II-III CRC after adjuvant chemotherapy.

## Introduction

Colorectal cancer (CRC) is well recognized for its clinical and biological diversities (1). Approximately three-fifths of CRC cases are stage II-III disease at diagnosis (2, 3). Radical resection is the preferred option for these patients (2–5). The treatment outcome of these patients remains unsatisfactory (2–6). Approximately 30% of CRC patients with stage II-III disease will experience tumor recurrence after radical resection (2–6). The clinical and biological diversities may present great challenges in identifying high-risk CRC patients, which subsequently makes it difficult to distinguish between CRC patients who may benefit from adjuvant chemotherapy when the probability of tumor recurrence is considered.

Macrophages are a main cellular component of the immune microenvironment (7). Tumor-associated macrophages may exhibit a spectrum of polarization status, with M_1_- and M_2_-macrophages representing the ends of this spectrum. Diametrically polarized macrophages may have opposite functions in tumor progression (7, 8). M_1_-macrophages may provide a resistant role in tumorigenesis by activating tumor-killing mechanisms and amplifying Th_1_ immunocyte responses (7, 8). However, M_2_-macrophages may stimulate tumor invasion and metastasis by suppressing tumor-specific immune responses (7, 8). Previous studies revealed that M_1_- and M_2_-macrophages exhibited high expression levels of CD86 and CD206 in gastrointestinal cancers, respectively (9–11). Therefore, we concluded that high infiltration of CD206^+^ macrophages, low infiltration of CD86^+^ macrophages, and a high ratio of CD206^+^/(CD86^+^+CD206^+^) macrophages would be markedly associated with advanced stage and a high rate of tumor recurrence and mortality (10–13). Actually, there are many tumor cases that may fall into a gray zone between M_1_- and M_2_-polarization. It is difficult to determine a suitable polarization phenotype for these cases. Thus, the tripartite categorization (M_1_-, mixed-, and M_2_-phenotype) may be a reasonable choice when one evaluates the polarization phenotype.

Integrating these immune markers into TNM staging might refine the prognostic significance for risk stratification and facilitate the development of better treatment strategies. Moreover, a single biomarker might not effectively characterize the complex immune microenvironment (14). In our study, we simultaneously assessed the infiltration intensity of stromal CD86^+^ and CD206^+^ macrophages by immunohistochemical staining. We developed two tripartite classification systems of CD86^+^ and CD206^+^ macrophages, namely, ratio and quantity subgroups, as prognostic tools for tumor recurrence and mortality. The first tripartite categorization, namely, ratio subgroups, was composed of the low-, moderate-, and high-ratio subgroups based on the lower- (LQ) and upper-quartile (UQ) cutoff points of the CD206^+^/(CD86^+^+CD206^+^) macrophage ratio, correspondingly representing the M_1_-, mixed-, and M_2_-phenotype. The secondary tripartite categorization, namely, quantity subgroups, consisted of the low-, moderate-, and high-risk subgroups determined by the median cutoff points of CD86^+^ and CD206^+^ macrophages infiltration density, correspondingly embodying the M_1_-, mixed-, and M_2_-phenotype.

## Materials and methods

### Study participants

We retrospectively collected 449 CRC cases with stage II-III disease from two different hospitals. Of 449 patients, 310 patients underwent radical operations at Yantai Yuhuangding Hospital of China between 2012 and 2015. The remaining 139 patients underwent radical operations at Guangzhou Red Cross Hospital of China between 2013 and 2015. All participants were restaged according to the 8th edition Staging Classification of American Joint Committee on Cancer. This protocol was authorized by the Ethics Committee of Yantai Yuhuangding Hospital (Approval No.2018-118) and Guangzhou Red Cross Hospital (Approval No.2019-227-01). The inclusion criteria for this study were as follows: (a) middle-high rectal cancer or colon cancer; (b) with paraffin-embedded tumor tissue and survival information; and (c) patients with stage II-III disease. The exclusion criteria for this study were as follows: (a) low rectal cancer; (b) without paraffin-embedded tumor tissue or survival information; (c) with secondary primary tumors before and at diagnosis; (d) patients with stage I or IV disease; and (e) with neoadjuvant chemotherapy, radiotherapy, and immunotherapy. The reason is that neoadjuvant treatment may affect the infiltration number of different polarized macrophages in tumoral tissues.

### Immunohistochemistry staining

The tumor sections (4 µm) for these 449 cases were collected for immunohistochemical staining of CD86 and CD206 in January 2020. Tumor sections were stained for CD86 and CD206 from February to April 2020. The Benchmark-XT immunohistochemistry platform (Roche Company, Switzerland) was adopted for immunohistochemistry staining according to the standard procedure. Anti-human CD86 (Catalog Number : DF6332, 1:200, Affinity, USA) and anti-human CD206 (Catalog Number:91992S, 1:400, CST, USA) primary antibodies were utilized for immunohistochemistry staining. The enzyme-labeled anti-mouse/rabbit polymerized secondary antibody (ready to use, Roche Company, Switzerland) was further adopted.

In colorectal cancer, tumor area consists of tumor nest and stroma (13). Macrophages mainly infiltrate in the tumor stroma (13). CD86^+^ or CD206^+^ macrophages in the tumor stroma were defined as those cells that stained brown. So only macrophages that infiltrated at the invasive margins of the tumor stroma were counted by three randomly selected fields (400×) under the Leica-DM-LB2 microsystem. Two experienced researchers were blinded to the clinicopathologic information, and independently assessed the infiltrating number of these three random fields at the invasive margins for each patient. The mean number of the two counting results was utilized for the infiltrating number of per field (400×). For each patient, the mean number of three random fields was further adopted for the infiltrating intensity of CD86^+^ or CD206^+^ macrophages. Intraobserver and inter-observer agreement was well acceptable (κ > 0.90).

### The definition of ratio and quantity subgroup systems

The first analysis was performed on the ratio subgroup system determined by the ratio of CD206^+^/(CD86^+^+CD206^+^) macrophages using the LQ and UQ cutoff points. The ratio subgroup system was composed of the low- (ratio≤LQ), moderate- (LQ<ratio≤UQ), and high-ratio (ratio>UQ) subgroups.

**Figure 1:**
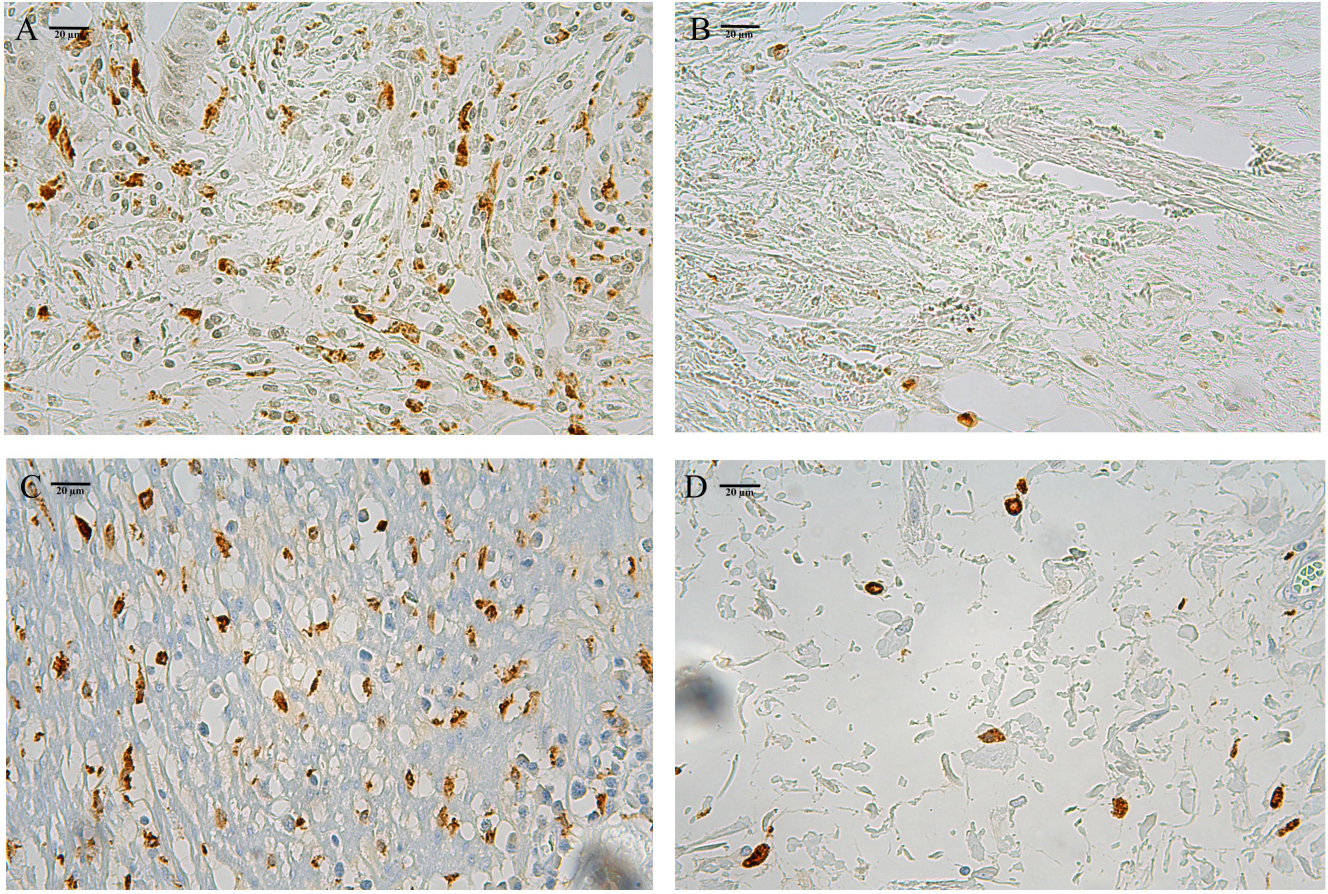
The high- and low-infiltration of stromal CD86^+^ **(A, B)** and CD206^+^ **(C, D)** macrophages.

The secondary analysis was performed on the quantity subgroup system based on the high- and low-infiltration groups of CD86^+^ (CD86^high^, **Figure 1A**; CD86^low^, **Figure 1B**) and CD206^+^ (CD206^high^, **Figure 1C**; CD206^low^, **Figure 1D**) macrophages using the median cutoff points. The quantity subgroup system was composed of the low- (CD86^high^/CD206^low^), moderate- (CD86^low^/CD206^low^ & CD86^high^/CD206^high^), and high-risk (CD86^low^/CD206^high^) subgroups.

### Exploration of macrophage-related gene sets based on microarray data

The microarray dataset GSE39582 was downloaded from the Gene Expression Omnibus repository, which was provided by the French national CIT program (15). The levels of gene expression were first normalized by the limma package and further log2-transformed. In this microarray dataset, fresh-frozen samples of primary tumor were collected for analyzing mRNA expression profiles by the GPL570 platform, including 460 stage II-III patients with complete clinical and survival information.

The CIBERSORT algorithm is an accurate tool for calculating the estimated proportion of M_1_- and M_2_-macrophages by imputing gene expression profiles of the microarray dataset GSE39582 (16). A value of *p*<0.05 is recommended for inclusion in the further analysis (16). And 379 cases with stage II-III disease were finally fitted in the subsequent analysis. The correlation of 21 immune cell types in CRC tissues (GSE39582) was evaluated by the correlation heatmap. This patient cohort was stratified into the low- and high-infiltration groups based on the optimal cutoffs of M_1_- or M_2_-macrophage proportions determined by the MaxStat method, separately (17).

To identify the enriched gene sets between high- and low-infiltration groups of M_1_- or M_2_-macrophages, we performed gene set enrichment analysis (GSEA) on all the mRNAs of the GPL570 platform using hallmark gene sets (18). We performed 1000 random sample permutations using the GSEA desktop application (version 4.3.0) to determine whether the members of a given gene set were associated with M_1_- or M_2_-macrophage infiltration. A threshold value of *p*<0.01 was considered significant.

### Statistical analysis

The chi-square test was utilized to evaluate the correlation between the tripartite categorizations and these clinicopathologic factors. A threshold value of *p*<0.05 was considered significant. The main endpoints included recurrence-free/overall survival (RFS/OS). The R software (version 4.2.1) was utilized for data analysis.

Kaplan-Meier analysis and log-rank test were utilized to assess survival differences among the three risk subgroups. Multivariate Cox analysis was used to determine whether ratio and quantity subgroups were independent of those clinicopathologic variables. Receiver operating characteristic (ROC) curves were performed to assess the prediction abilities of tumor stage, ratio and quantity subgroups (19). To construct ROC curves by the pROC package (19), patients with a duration of ≤48 months were excluded if they still did not experience tumor recurrence at the final follow-up. The recurrence-free time of the remaining cases was divided into either ≤48 months or >48 months.

## Results

### Prognostic values of the ratio subgroup system

#### Survival differences among the three ratio subgroups

**Figure 2:**
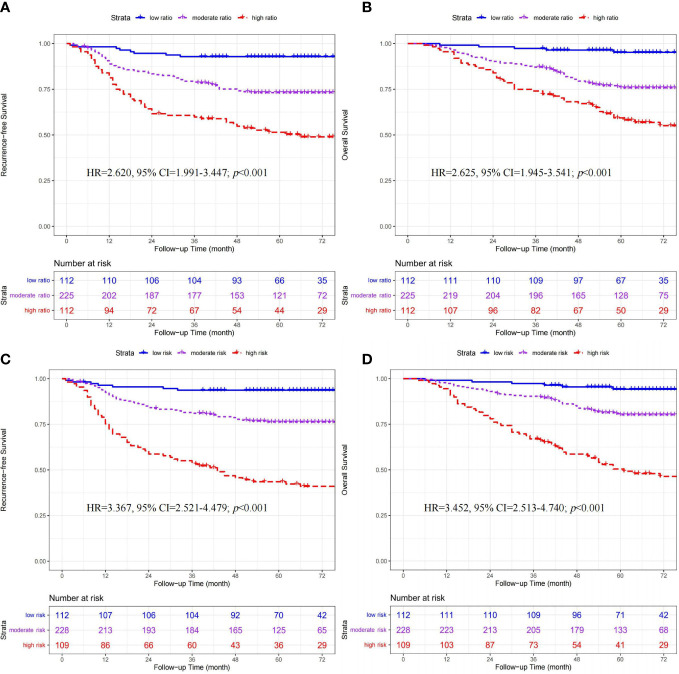
Kaplan-Meier curves of recurrence-free survival and overall survival stratified by ratio **(A, B)** and quantity **(C, D)** subgroups in 449 CRC patients.

The ratio of CD206^+^/(CD206^+^+CD86^+^) macrophages ranged from 0.019 to 0.993. Based on the lower- (0.285) and upper-quartile (0.709) points, 449 patients were stratified into the low- (n=112), moderate- (n=225), and high-ratio (n=112) subgroups. Clinicopathologic factors among the three ratio subgroups are shown in **Table 1**. As shown in **Figures 2A, B**, the ratio subgroup system (high- *vs.* moderate- *vs.* low-ratio) was significantly associated with worse RFS (hazard ratio [HR]=2.620, 95% confidence interval [CI]=1.991-3.447; *p*<0.001) and OS (HR=2.625, 95% CI=1.945-3.541; *p*<0.001).

**Table 1:** Clinical and pathological characteristics of 449 CRC patients among different risk subgroups.

Variable	Ratio Subgroups				Quantity Subgroups			
	low ratio (n=112)	moderate ratio (n=225)	high ratio (n=112)	*p*.value	low risk (n=112)	moderate risk (n=228)	high risk (n=109)	*p*.value
Age								
<66 y	53	115	70	0.054	63	115	60	0.533
≥66 y	59	110	42		49	113	49	
Gender								
male	69	124	69	0.377	70	130	62	0.590
female	43	101	43		42	98	47	
Mucinous Component								
no	106	196	100	0.104	108	200	94	0.021
yes	6	29	12		4	28	15	
Primary Locations								
colon	53	110	41	0.092	45	115	44	0.096
rectum	59	115	71		67	113	65	
Tumor Stage								
II	55	111	40	0.045	63	108	35	0.001
III	57	114	72		49	120	74	
Adjuvant Chemotherapy								
yes	74	146	74	<0.001	77	144	73	<0.001
no	38	79	38		35	84	36	

#### Multivariate COX analysis of ratio subgroups and other clinicopathologic factors

Multivariate analysis demonstrated that the ratio subgroup system remained an independent factor for RFS (HR=2.677, 95% CI=2.028-3.533; *p*<0.001) and OS (HR=2.708, 95% CI=1.998-3.670; *p*<0.001) (**Table 2**).

**Table 2:** Multivariate Cox analysis of macrophage-based risk subgroups, clinicopathologic factors and survival.

Variable	Recurrence-free Survival		Overall Survival	
	HR (95% CI)	p value	HR (95% CI)	p value
Ratio Subgroups				
ratio subgroups (high vs. moderate vs. low ratio)	2.677 (2.028-3.533)	<0.001	2.708 (1.998-3.670)	<0.001
age (≥66 y vs. <66 y)	1.337 (0.910-1.964)	0.139	1.743 (1.145-2.654)	0.010
gender (female vs. male)	1.212 (0.843-1.742)	0.299	1.067 (0.716-1.588)	0.753
mucinous component (yes vs. no)	0.861 (0.448-1.656)	0.655	0.851 (0.410-1.766)	0.665
primary locations (rectum vs. colon)	0.738 (0.508-1.072)	0.111	0.748 (0.500-1.119)	0.158
tumor stage (III vs. II)	3.345 (2.174-5.146)	<0.001	3.580 (2.218-5.779)	<0.001
adjuvant chemotherapy (no vs. yes)	0.756 (0.506-1.132)	0.174	0.779 (0.508-1.197)	0.255
Quantity Subgroups				
quantity subgroups (high vs. moderate vs. low risk)	3.137 (2.342-4.200)	<0.001	3.250 (2.357-4.483)	<0.001
age (≥66 y vs. <66 y)	1.086 (0.743-1.589)	0.670	1.455 (0.962-2.201)	0.076
gender (female vs. male)	1.095 (0.764-1.569)	0.622	0.949 (0.639-1.409)	0.796
mucinous component (yes vs. no)	0.693 (0.359-1.338)	0.275	0.678 (0.326-1.414)	0.300
primary locations (rectum vs. colon)	0.792 (0.547-1.147)	0.217	0.792 (0.530-1.181)	0.253
tumor stage (III vs. II)	2.804 (1.821-4.317)	<0.001	3.088 (1.914-4.984)	<0.001
adjuvant chemotherapy (no vs. yes)	0.788 (0.527-1.179)	0.247	0.792 (0.515-1.217)	0.287

HR, hazard ratio, CI, confidence interval.

#### The prognostic value of ratio subgroups for CRC patients receiving adjuvant chemotherapy

**Figure 3:**
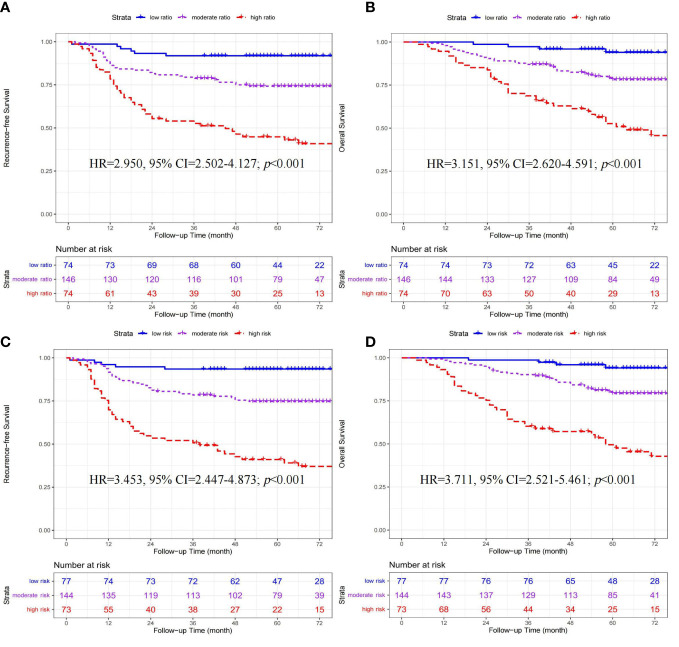
Kaplan-Meier curves of recurrence-free survival and overall survival stratified by ratio **(A, B)** and quantity **(C, D)** subgroups in 294 CRC patients receiving adjuvant chemotherapy.

In 294 patients receiving chemotherapy (Shown in **Figures 3A, B**), significant differences in RFS (HR=2.950, 95% CI=2.502-4.127; *p*<0.001) and OS (HR=3.151, 95% CI=2.620-4.591; *p*<0.001) were found among the three ratio subgroups (high- *vs.* moderate- *vs.* low-ratio).

### Prognostic values of the quantity subgroup system

#### Survival differences among the three quantity subgroups

According to the median cutoff points, 449 patients were classified into three quantity subgroups, including the low- (CD86^high^/CD206^low^, n=112), moderate- (CD86^low^/CD206^low^ & CD86^high^/CD206^high^, n=228), and high-risk (CD86^low^/CD206^high^, n=109) subgroups. The clinicopathologic characteristics among the three ratio subgroups are presented in **Table 1**. As shown in **Figures 2C, D**, the quantity subgroup system (high- *vs.* moderate- *vs.* low-risk) was significantly correlated with worse RFS (HR=3.367, 95% CI=2.521-4.479; *p*<0.001) and OS (HR=3.452, 95% CI=2.513-4.740; *p*<0.001).

#### Multivariate COX analysis of quantity subgroups and other clinicopathologic factors

Multivariate analysis demonstrated that the quantity subgroup system remained an independent indicator for RFS (HR=3.137, 95% CI=2.342-4.200; *p*<0.001) and OS (HR=3.250, 95% CI=2.357-4.483; *p*<0.001) (**Table 2**).

#### The prognostic value of quantity subgroups for CRC patients receiving adjuvant chemotherapy

In 294 patients receiving chemotherapy (Shown in **Figures 3C, D**), significant differences in RFS (HR=3.453, 95% CI=2.447-4.873; *p*<0.001) and OS (HR=3.711, 95% CI=2.521-5.461; *p*<0.001) were found among the three quantity subgroups (high- *vs.* moderate- *vs.* low-risk).

### ROC curve analysis for ratio and quantity subgroups

**Figure 4:**
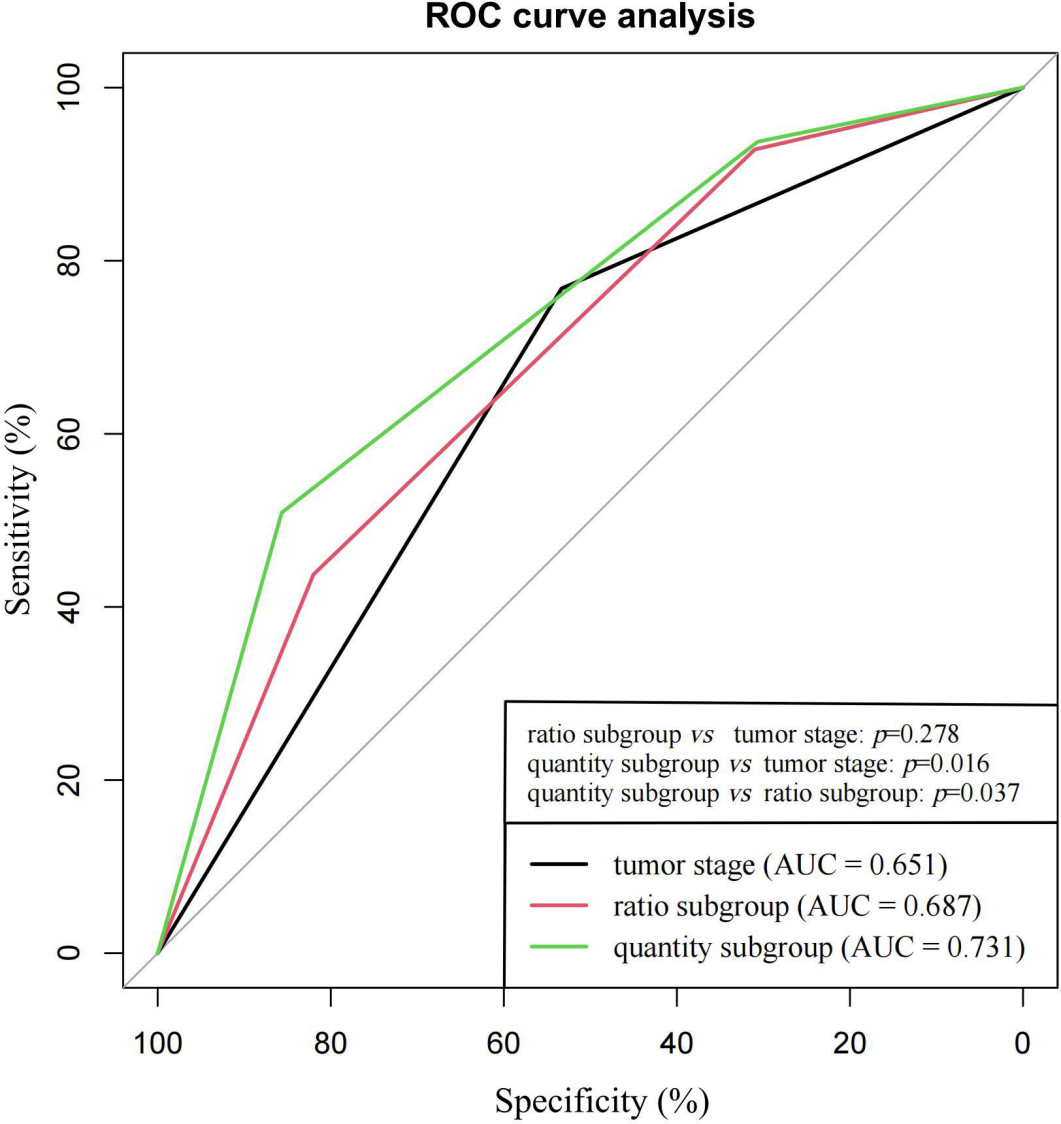
ROC curve analysis for ratio subgroups, quantity subgroups and tumor stage in the prediction of tumor recurrence within 48 months.

As shown in **Figure 4**, the predictive accuracy of the quantity subgroups within 48 months was higher than that of the ratio subgroups (area under the curve [AUC]: 0.731 *vs.* 0.687, *p*=0.037) and tumor stage (AUC: 0.731 *vs.* 0.651, *p*=0.016). Despite the lack of significant difference, the predictive accuracy of the ratio subgroups tended to be higher than that of tumor stage (AUC: 0.687 *vs.* 0.651, *p*=0.278).

### Integrated analysis of ratio and quantity subgroups

**Figure 5:**
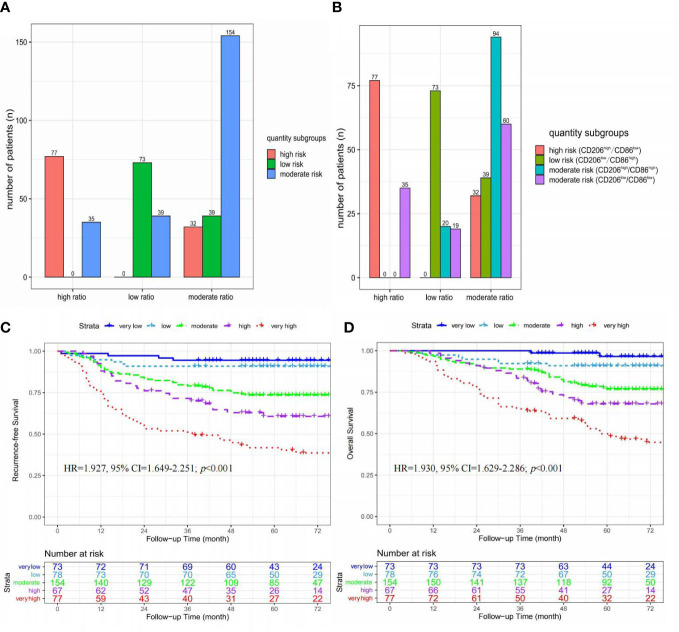
The integrated analysis of ratio and quantity subgroups. **(A)** Histograms of the relationship between ratio and quantity subgroups; **(B)** Histograms of the relationship between ratio subgroups and immunohistochemistry results; **(C)** Kaplan-Meier curves of recurrence-free survival stratified by five risk subgroups; **(D)** Kaplan-Meier curves of overall survival stratified by five risk subgroups.

As shown in **Figures 5A, B**, 304 (66.7%) of 449 patients had an evaluation of the same risk level according to two tripartite categorization systems. Among these 304 patients, there were 73 patients with a low risk of tumor recurrence in both the low-ratio and low-risk subgroups, 154 patients with a moderate risk of tumor recurrence in both the moderate-ratio and moderate-risk subgroups, and 77 patients with a high risk of tumor recurrence in both the high-ratio and high-risk subgroups.

These 449 patients were stratified into five risk subgroups based on an integrated analysis of ratio and quantity subgroups, including very low (low-risk & low-ratio, n=73), low (moderate-risk & low-ratio, n=39; low-risk & moderate-ratio, n=39), moderate (moderate-risk & moderate-ratio, n=154), high (high-risk & moderate-ratio, n=32; moderate-risk & high-ratio, n=35), and very high (high-risk & high-ratio, n=35) risk subgroups (**Figures 5A, B**).

As shown in **Figures 5C, D**, significant differences in RFS (HR=1.927, 95% CI=1.649-2.251; *p*<0.001) and OS (HR=1.930, 95% CI=1.629-2.286; *p*<0.001) were found among these five risk subgroups (very-high *vs.* high *vs.* moderate *vs.* low *vs.* very-low).

### Macrophage-related gene sets based on microarray data analysis

#### The M_1_-related gene sets

**Figure 6:**
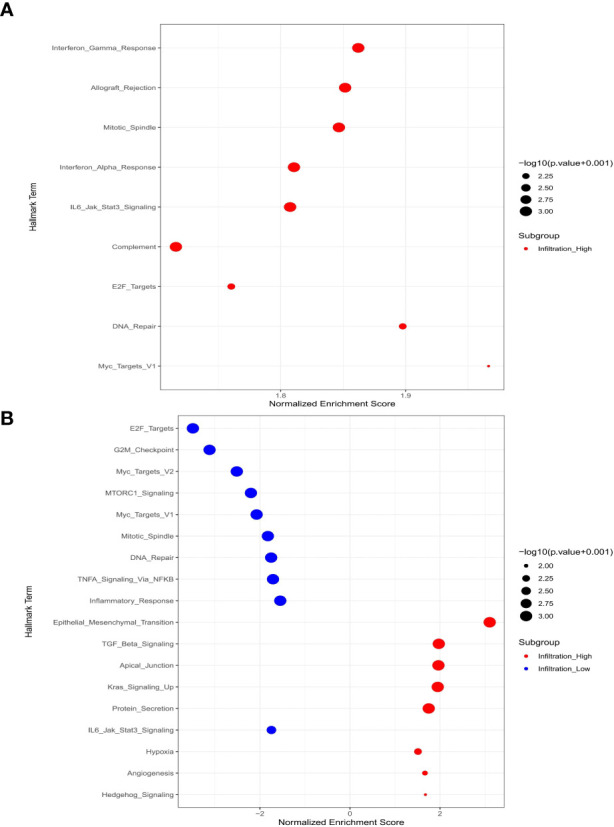
Macrophage-related signal pathways based on microarray data analysis. **(A)** M_1_-macrophages; **(B)** M_2_-macrophages.

**Figure 7:**
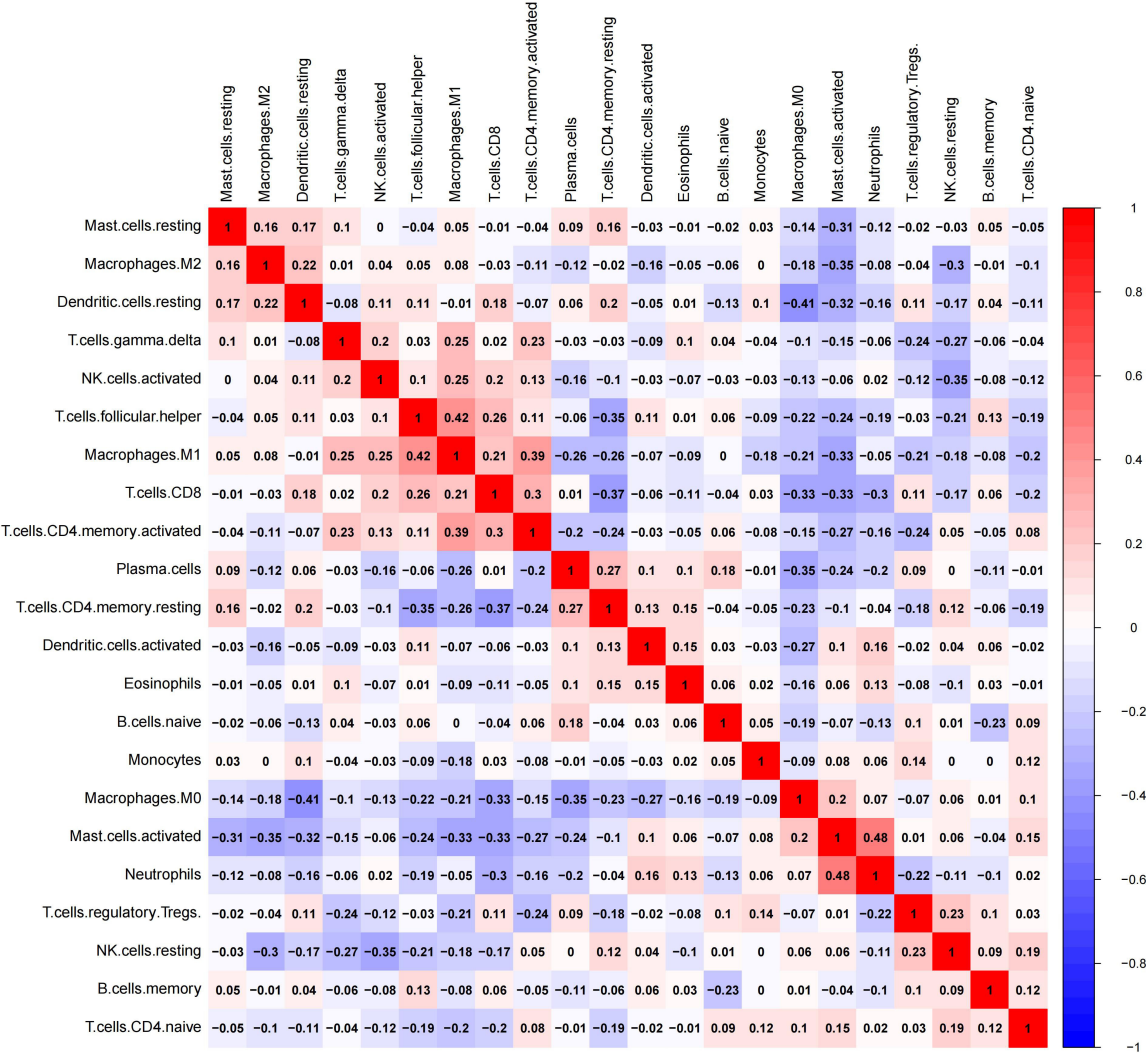
The correlation heatmap of 21 immune cell types in patients with stage II-III CRC.

The infiltration of M_1_-macrophages was enriched in nine gene sets for the high-infiltration group (Shown in **Figure 6A**, **Supplementary Table S1**). No gene set was significantly enriched in the low M_1_-infiltration group. The GSEA results implied that the interferon-α response, mitotic spindle, IL6/Jak/Stat3 signaling, E2F targets, allograft rejection, DNA repair, myc targets V1, complement, and interferon-γ response pathways were significantly correlated with the high M_1_-infiltration. The relationship between M_1_-macrophages and other immune cell subtypes was shown in **Figure 7**.

#### The M_2_-related gene sets

The infiltration of M_2_ macrophages was involved in eight gene sets for the high-infiltration group and ten gene sets for the low-infiltration group (Shown in **Figure 6B**, **Supplementary Table S1**). We observed that epithelial mesenchymal transition, TGF-β signaling, apical junction, kras signaling up, protein secretion, hypoxia, angiogenesis, and hedgehog signaling pathways were significantly correlated with the high M_2_-infiltration group. The GSEA results implied that the mitotic spindle, E2F targets, IL6/Jak/Stat3 signaling, DNA repair, G2-M checkpoint, myc targets V2, MTORC1 signaling, myc targets V1, inflammatory response, and TNF-α/NF-KB signaling pathways were significantly associated with the low M_2_-infiltration group. The relationship between M_2_-macrophages and other immune cell subtypes was shown in **Figure 7**.

## Discussion

Although the TNM staging system can provide essential prognostic information for determining therapeutic regimens, it does not incorporate any immune microenvironment information into the staging algorithm. It’s noted that even CRC patients with the same stage might exhibit conflicting results (2–6). In our study, we constructed the tripartite classifications of ratio and quantity subgroups by the integrated analysis of CD86^+^ and CD206^+^ macrophages. And the ratio and quantity subgroups could effectively stratify these CRC patients with stage II-III disease into three risk groups with a low-, moderate-, and high-risk of tumor recurrence. The ratio and quantity subgroups could effectively predict treatment recurrence and mortality independent of tumor stage and other clinicopathologic factors. Compared with ratio subgroups and tumor stage, quantity subgroups may have the optimal prediction ability of tumor recurrence within 48 months. Based on the further combined analysis of ratio and quantity subgroups, stage II-III CRC could be stratified into five risk subgroups (very-high, high, moderate, low, and very-low) with significant differences in RFS and OS.

At present, tumor stage and the clinicopathologic factors remain the most important factors in the decision-making process of adjuvant chemotherapy. The 5-year survival benefit of postoperative chemotherapy is only 2%-5% for stage II CRC (20–22). And the assessment system of recurrent risk should be improved to optimize the treatment strategy in stage II CRC. In clinical practice, all CRC patients with stage III disease might be unreasonably given the long duration (six months) of postoperative chemotherapy indistinguishably (23–25). In our study, the log-rank test revealed that CRC patients in the high-ratio or high-risk subgroup exhibited the worst RFS and OS, CRC patients in the low-ratio or low-risk subgroup exhibited the optimal RFS and OS after adjuvant chemotherapy. These two tripartite classifications might enable medical oncologists to precisely stratify stage II-III CRC for avoiding overtreatment or undertreatment in some specific patients.

Tumor-associated macrophages exhibit a spectrum of polarization status, with M_1_- and M_2_-macrophages representing the ends of this spectrum. A meta-analysis of 29 studies demonstrated that high infiltration of CD68^+^ pan-macrophages at invasive margins was significantly associated with better survival, while high infiltration of M_2_-macrophages in tumor center was significantly associated with poor prognosis in CRC patients (26). Nevertheless, the clinical significance of M_1_-macrophages in CRC was still controversial (26). Actually, most macrophages belong to a mixed M_1_/M_2_ phenotype (27, 28). The combined analysis of M_1_- and M_2_-macrophages provides more comprehensive information on tumor prognosis. Yang et al. demonstrated that high ratio of CD163^+^/CD68^+^ macrophages was significantly associated with poor prognosis in patients with CRC (29). Feng et al. found that high ratio of CD206^+^/CD68^+^ macrophages was significantly associated with poor survival and could be used for a better predictive biomarker for adjuvant chemotherapy in stage II CRC (13). In this study, CRC patients in the moderate-risk/moderate-ratio subgroup had intermediate RFS and OS, and these patients exhibited a relatively functional counterbalance regulated by M_1_- and M_2_-macrophages. The low-risk/low-ratio subgroup represented a polarization profile of M_1_-macrophages and were associated with a favorable prognosis. The GSEA results implied that the tumor-killing mechanism of M_1_-macrophages was potentially derived from immune activation of the IFN-α (30) and IFN-γ (31) response pathways. However, the high-risk/high-ratio subgroups might demonstrate a polarization profile of M_2_-macrophages and serve as a poor prognosis. The GSEA results also implied that the protumor mechanism of M_2_-macrophages might be achieved by activating the epithelial mesenchymal transition (32), TGF-β signaling (33), hedgehog signaling (34), angiogenesis (35), and hypoxia (36) pathways. These findings might further confirm the opposite functions of diametrically polarized macrophages.

The ratio and quantity subgroups might complement each other in stage II-III CRC. Firstly, 35 CRC cases were classified into both the high-ratio (ratio>p75) and moderate-risk (CD86^low^/CD206^low^) subgroups. The quantity subgroups might be more scientific and credible for these patients with a moderate risk of postoperative recurrence. Secondly, 19 CRC cases in both the low-ratio (ratio≤p25) and moderate-risk (CD86^low^/CD206^low^) subgroups might have a moderate risk of postoperative recurrence. The quantity subgroups could contribute to defining the actual risk of these 19 cases. Thirdly, 20 CRC cases in both the low-ratio (ratio≤p25) and moderate-risk (CD86^high^/CD206^high^) subgroups might have a low risk of postoperative recurrence. The ratio subgroups could contribute to defining the actual risk of these 20 cases. Fourthly, 32 CRC cases in the low-risk (CD86^high^/CD206^low^) subgroup and 39 CRC cases in the high-risk (CD86^low^/CD206^high^) subgroup were classified into the moderate-ratio (p25<ratio≤p75) subgroup. Most of these CRC cases might exhibit a mixed M_1_/M_2_ phenotype with an intermediate risk of postoperative recurrence.

In addition to their prognostic roles, growing evidences have revealed that macrophages represent a new anticancer target (37, 38). As a selective inhibitor of CSF1R kinase, GW2580 reduces M_2_-macrophage infiltration and normalizes the disorganized peritoneal vasculature in GW2580-treated ascites of ovarian cancer (37). GW2580 also enhances the anticancer and antiangiogenic effects of an anti-VEGFR-2 antibody in mouse tumor models (38). As a synthetic vitamin A derivative, fenretinide suppresses M_2_-macrophages by inhibiting STAT6 phosphorylation and further preventing the tumorigenesis of colon carcinoma (39). IFN-γ recovers the M_1_-phenotype through the increased expression of CD86, enhancement of the infiltration of cytotoxic T cells, and the transformation of an immunosuppressive phenotype into an immunostimulatory phenotype in IFN-γ-treated ascites of ovarian cancer (40). These findings imply that macrophage-targeted therapy may represent a promising strategy.

This study has several potential limitations. Firstly, our study design was retrospective. The prognostic significance of chemotherapy regimens and cycle was not included in the analysis. Secondly, as an unresolved issue (41), the semi-quantitative method for immunohistochemical staining might not completely reflect the actual intensity of macrophage infiltration. Thirdly, this is also an unresolved issue for the immune cells with a very accurate marker of immunohistochemical staining. Although most of M1-macrophages expressed this biomarker of CD86, it doesn’t mean that all CD86^+^ cells are M1-macrophages (42, 43). And a small proportion of CD86^+^ cells might belong to M2b macrophages and other immune cells (42–44). So far, there is no study of colorectal cancer to explore the expression difference of CD206 in different macrophage subtypes (M2a、M2b and M2c). According to the previous study results of macrophage polarization (13, 45, 46), we adopted CD86 and CD206 to work as the biomarkers for M1- and M2-macrophages, respectively.

In conclusion, both ratio and quantity subgroups effectively stratify CRC patients with stage II-III disease into three subgroups with a low, moderate, and high risk of treatment relapse and mortality. Compared with ratio subgroups and tumor stage, quantity subgroups could more effectively predict treatment recurrence with 48 months. In addition, immunohistochemical staining of CD86^+^ and CD206^+^ macrophages is easy, inexpensive and rapid and can be performed in most hospitals. Upon further assessment in multiple-center prospective studies, these prognostic biomarkers of ratio and quantity subgroups will contribute to the implementation of precision treatment strategies for stage II-III CRC.

## Data availability statement

The original contributions presented in the study are included in the article/**Supplementary Material**. Further inquiries can be directed to the corresponding authors.

## Ethics statement

This study was authorized by the Ethics Committee of Yantai Yuhuangding Hospital (Shandong, China) and Guangzhou Red Cross Hospital (Guangdong, China). The patients/participants provided their written informed consent to participate in this study.

## Author contributions

FZ, JC, and GX conceived and designed this study. GX, JL, and YM performed sample detection and the experiments. GX and YM collected the data and performed the statistical analysis. GX and QW carried out this manuscript. All authors contributed to the article and approved the submitted version.

## Supplementary material

Supplementary Table 1 The GSEA results of macrophage-related pathways based on microarray data analysis. NES, Normalized Enrichment Score, FDR, False Discovery Rate.

## References

[1] Sveen A Kopetz S Lothe RA. Biomarker-guided therapy for colorectal cancer: strength in complexity. Nat Rev Clin Oncol (2020) 17:11–32. doi: 10.1038/s41571-019-0241-1 31289352 PMC7577509

[2] Giráldez MD Lozano JJ Cuatrecasas M Alonso-Espinaco V Maurel J Marmol M. Gene-expression classifier of tumor recurrence in patients with stage II and III colon cancer treated with 5'fluoruracil-based adjuvant chemotherapy. Int J Cancer (2013) 132:1090–7. doi: 10.1002/ijc.27747 22833293

[3] André T Boni C Mounedji-Boudiaf L Tabernero J Hickish J Topham C. Oxaliplatin, fluorouracil, and leucovorin as adjuvant treatment for colon cancer. N Engl J Med (2004) 350:2343–51. doi: 10.1056/NEJMoa032709 15175436

[4] Yothers G O'Connell MJ Allegra CJ Kuebler JP Colangelo LH Petrelli NJ. Oxaliplatin as adjuvant therapy for colon cancer: updated results of NSABP c-07 trial, including survival and subset analyses. J Clin Oncol (2011) 29:3768–74. doi: 10.1200/JCO.2011.36.4539 PMC3188282 21859995

[5] Haller DG Tabernero J Maroun J de Braud F Price T Van Cutsem E. Capecitabine plus oxaliplatin compared with fluorouracil and folinic acid as adjuvant therapy for stage III colon cancer. J Clin Oncol (2011) 29:1465–71. doi: 10.1200/JCO.2010.33.6297 21383294

[6] Quasar Collaborative Group Quasar Collaborative Group Gray R Barnwell J McConkey C Hills RK Williams NS Kerr DJ. Adjuvant chemotherapy versus observation in patients with colorectal cancer: a randomised study. Lancet (2007) 370:2020–9. doi: 10.1016/S0140-6736(07)61866-2 18083404

[7] Murray PJ Wynn TA. Protective and pathogenic functions of macrophage subsets. Nat Rev Immunol (2011) 11:723–37. doi: 10.1038/nri3073 PMC3422549 21997792

[8] Heusinkveld M vander Burg SH. Identification and manipulation of tumor associated macrophages in human cancers. J Transl Med (2011) 9:216. doi: 10.1186/1479-5876-9-216 22176642 PMC3286485

[9] Hernández C Barrachina MD Cosín-Roger J Ortiz-Masia D Álvarez Á Terradez L. Progastrin represses the alternative activation of human macrophages and modulates their influence on colon cancer epithelial cells. PloS One (2014) 9:e98458. doi: 10.1371/journal.pone.0098458 24901518 PMC4047028

[10] Zhu F Li X Jiang Y Zhu H Zhang H Zhang C. GdCl3 suppresses the malignant potential of hepatocellular carcinoma by inhibiting the expression of CD206 in tumor-associated macrophages. Oncol Rep (2015) 34:2643–55. doi: 10.3892/or.2015.4268 26352004

[11] Kim HD Kim SY Kim J Kim JE Hong YS Han B. Dynamic increase of M2 macrophages is associated with disease progression of colorectal cancers following cetuximab-based treatment. Sci Rep (2022) 12:1678. doi: 10.1038/s41598-022-05694-x 35102212 PMC8803829

[12] Kou Y Li Z Sun Q Yang S Wang Y Hu C. Prognostic value and predictive biomarkers of phenotypes of tumour-associated macrophages in colorectal cancer. Scand J Immunol (2022) 95:e13137. doi: 10.1111/sji.13137 34964155 PMC9286461

[13] Feng Q Chang W Mao Y He G Zheng P Tang W. Tumor-associated macrophages as prognostic and predictive biomarkers for postoperative adjuvant chemotherapy in patients with stage II colon cancer. Clin Cancer Res (2019) 25(13):3896–907. doi: 10.1158/1078-0432.CCR-18-2076 30988081

[14] Ishigami S Natsugoe S Tokuda K Nakajo A Okumura H Matsumoto M. Tumor-associated macrophage (TAM) infiltration in gastric cancer. Anticancer Res (2003) 5A:4079–83. 14666722

[15] Marisa L de Reyniès A Duval A Selves J Gaub MP Vescovo L. Gene expression classification of colon cancer into molecular subtypes: characterization, validation, and prognostic value. PloS Med (2013) 10:e1001453. doi: 10.1371/journal.pmed.1001453 23700391 PMC3660251

[16] Chen B Khodadoust MS Liu CL Newman AM Alizadeh AA. Profiling tumor infiltrating immune cells with CIBERSORT. Methods Mol Biol (2018) 1711:243–59. doi: 10.1007/978-1-4939-7493-1-12 PMC5895181 29344893

[17] Ogłuszka M Orzechowska M Jędroszka D Witas P Bednarek AK. Evaluate cutpoints: adaptable continuous data distribution system for determining survival in Kaplan-Meier estimator. Comput Methods Programs BioMed (2019) 177:133–9. doi: 10.1016/j.cmpb.2019.05.023 31319941

[18] Subramanian A Tamayo P Mootha VK Mukherjee S Ebert BL Gillette MA. Gene set enrichment analysis: a knowledge-based approach for interpreting genome-wide expression profiles. Proc Natl Acad Sci (2005) 102:15545–50. doi: 10.1073/pnas.0506580102 PMC1239896 16199517

[19] Robin X Turck N Hainard A Tiberti N Lisacek F Sanchez JC. pROC: an open-source package for r and s+ to analyze and compare ROC curves. BMC Bioinf (2011) 12:77. doi: 10.1186/1471-2105-12-77 PMC3068975 21414208

[20] Iveson TJ Sobrero AF Yoshino T Youglakos I Ou FS Meyers JP. Duration of adjuvant doublet chemotherapy (3 or 6 months) in patients with high-risk stage II colorectal cancer. J Clin Oncol (2021) 39:631–41. doi: 10.1200/JCO.20.01330 PMC8078416 33439695

[21] Yamazaki K Yamanaka T Shiozawa M Manaka D Kotaka M Gamoh M. Oxaliplatin-based adjuvant chemotherapy duration (3 versus 6 months) for high-risk stage II colon cancer: the randomized phase III ACHIEVE-2 trial. Ann Oncol (2021) 32:77–84. doi: 10.1016/j.annonc.2020.10.480 33121997

[22] O'Connor ES Greenblatt DY LoConte NK Gangnon RE Liou JI Heise CP. Adjuvant chemotherapy for stage II colon cancer with poor prognostic features. J Clin Oncol (2011) 29:3381–8. doi: 10.1200/JCO.2010.34.3426 PMC3164243 21788561

[23] André T Meyerhardt J Iveson T Sobrero A Yoshino T Souglakos I. Effect of duration of adjuvant chemotherapy for patients with stage III colon cancer (IDEA collaboration): final results from a prospective, pooled analysis of six randomised, phase 3 trials. Lancet Oncol (2020) 21:1620–29. doi: 10.1016/S1470-2045(20)30527-1 PMC7786835 33271092

[24] Twelves C Wong A Nowacki MP Abt M Burris H 3rd Carrato A. Capecitabine as adjuvant treatment for stage III colon cancer. N Engl J Med (2005) 352:2696–704. doi: 10.1056/NEJMoa043116 15987918

[25] Grothey A Sobrero AF Shields AF Yoshino T Paul J Taieb J. Duration of adjuvant chemotherapy for stage III colon cancer. N Engl J Med (2018) 378:1177–88. doi: 10.1056/NEJMoa1713709 PMC6426127 29590544

[26] Yang Z Zhang M Peng R Liu J Wang F Li Y. The prognostic and clinicopathological value of tumor-associated macrophages in patients with colorectal cancer: a systematic review and meta-analysis. Int J Colorectal Dis (2020) 35:1651–61. doi: 10.1007/s00384-020-03686-9 32666290

[27] Gao J Liang Y Wang L. Shaping polarization of tumor-associated macrophages in cancer immunotherapy. Front Immunol (2022) 13:888713. doi: 10.3389/fimmu.2022.888713 35844605 PMC9280632

[28] Gunaydin G. CAFs interacting with TAMs in tumor microenvironment to enhance tumorigenesis and immune evasion. Front Oncol (2021) 11:668349. doi: 10.3389/fonc.2021.668349 34336660 PMC8317617

[29] Yang C Wei C Wang S Shi D Zhang C Li X. Elevated CD163+/CD68+ ratio at tumor invasive front is closely associated with aggressive phenotype and poor prognosis in colorectal cancer. Int J Biol Sci (2019) 15:984–98. doi: 10.7150/ijbs.29836 PMC6535793 31182919

[30] Belardelli F Ferrantini M Proietti E Kirkwood JM. Interferon-alpha in tumor immunity and immunotherapy. Cytokine Growth Factor Rev (2002) 13:119–34. doi: 10.1016/s1359-6101(01)00022-3 11900988

[31] Kim OY Park HT Dinh NTH Choi SJ Lee J Kim JH. Bacterial outer membrane vesicles suppress tumor by interferon-γ-mediated antitumor response. Nat Commun (2017) 8:626. doi: 10.1038/s41467-017-00729-8 28931823 PMC5606984

[32] Xuefeng X Hou MX Yang ZW Agudamu A Wang F Su XL. Epithelial-mesenchymal transition and metastasis of colon cancer cells induced by the FAK pathway in cancer-associated fibroblasts. J Int Med Res (2020) 48:300060520931242. doi: 10.1177/0300060520931242. 300060520931242. PMC7323289 32588696

[33] Tauriello DVF Palomo-Ponce S Stork D Berenguer-Llergo A Badia-Ramentol J Iglesias M. TGF-β drives immune evasion in genetically reconstituted colon cancer metastasis. Nature (2018) 554:538–43. doi: 10.1038/nature25492 29443964

[34] Skoda AM Simovic D Karin V Kardum V Vranic S Serman L. The role of the hedgehog signaling pathway in cancer: a comprehensive review. Bosn J Basic Med Sci (2018) 18:8–20. doi: 10.17305/bjbms.2018.2756 29274272 PMC5826678

[35] Zheng X Liu J Li X Tian R Shang K Dong X. Angiogenesis is promoted by exosomal DPP4 derived from 5-fluorouracil-resistant colon cancer cells. Cancer Lett (2021) 497:190–201. doi: 10.1016/j.canlet.2020.10.009 33039561

[36] Liu X Wan X Kan H Wang Y Yu F Feng L. Hypoxia-induced upregulation of Orai1 drives colon cancer invasiveness and angiogenesis. Eur J Pharmacol (2018) 832:1–10. doi: 10.1016/j.ejphar.2018.05.008 29753044

[37] Moughon DL He H Schokrpur S Jiang ZK Yaqoob M David J. Macrophage blockade using CSF1R inhibitors reverses the vascular leakage underlying malignant ascites in late-stage epithelial ovarian cancer. Cancer Res (2015) 75(22):4742–52. doi: 10.1158/0008-5472.CAN-14-3373 PMC4675660 26471360

[38] Priceman SJ Sung JL Shaposhnik Z Burton JB Torres-Collado AX Moughon DL. Targeting distinct tumor-infiltrating myeloid cells by inhibiting CSF-1 receptor: combating tumor evasion of antiangiogenic therapy. Blood (2010) 115:1461–71. doi: 10.1182/blood-2009-08-237412 PMC2826767 20008303

[39] Dong R Gong Y Meng W Yuan M Zhu H Ying M. The involvement of M2 macrophage polarization inhibition in fenretinide-mediated chemopreventive effects on colon cancer. Cancer Lett (2017) 388:43–53. doi: 10.1016/j.canlet.2016.11.029 27913199

[40] Duluc D Corvaisier M Blanchard S Catala L Descamps P Gamelin E. Interferon-γ reverses the immunosuppressive and protumoral properties and prevents the generation of human tumor-associated macrophages. Int J Cancer (2009) 125:367–73. doi: 10.1002/ijc.24401 19378341

[41] Schalper KA Brown J Carvajal-Hausdorf D McLaughlin J Velcheti V Syrigos KN. Objective measurement and clinical significance of TILs in non-small cell lung cancer. J Natl Cancer Inst (2015) 107:dju435. doi: 10.1093/jnci/dju435 25650315 PMC4565530

[42] Dugast AS Haudebourg T Coulon F Heslan M Haspot F Poirier N. Myeloid-derived suppressor cells accumulate in kidney allograft tolerance and specifically suppress effector T cell expansion. J Immunol (2008) 180:7898–906. doi: 10.4049/jimmunol.180.12.7898 18523253

[43] Dolen Y Gunaydin G Esendagli G Guc D. Granulocytic subset of myeloid derived suppressor cells in rats with mammary carcinoma. Cell Immunol (2015) 295:29–35. doi: 10.1016/j.cellimm.2015.02.005 25732602

[44] Wang LX Zhang SX Wu HJ Rong XL Guo J. M2b macrophage polarization and its roles in diseases. J Leukoc Biol (2019) 106(2):345–58. doi: 10.1002/JLB.3RU1018-378RR PMC7379745 30576000

[45] Dong P Ma L Liu L Zhao G Zhan S Dong L. CD86+/CD206+, diametrically polarized tumor-associated macrophages, predict hepatocellular carcinoma patient prognosis. Int J Mol Sci (2016) 17:320. doi: 10.3390/ijms17030320 26938527 PMC4813183

[46] Sun D Luo T Dong P Zhang N Chen J Zhang S. CD86(+)/CD206(+) tumor-associated macrophages predict prognosis of patients with intrahepatic cholangiocarcinoma. Peer J (2020) 8:e8458. doi: 10.7717/peerj.8458 32002338 PMC6982414

