# Supplementary material for: Two tripartite classification systems of CD86+ and CD206+ macrophages are significantly associated with tumor recurrence in stage II-III colorectal cancer
Source: Front Immunol. 2023 Jun 5;14:1136875. doi: 10.3389/fimmu.2023.1136875 (PMC10277500; doi:10.3389/fimmu.2023.1136875)
Supplement: Supplementary Table 1 — The GSEA results of macrophage-related pathways based on microarray data analysis. NES, Normalized Enrichment Score, FDR, False Discovery Rate. [file Table_1.docx]

| **Analysis Items** | **Description** | **Set Size** | **NES** | ***p*.value** | **FDR q-value** |
| --- | --- | --- | --- | --- | --- |
| M1-high Related Gene Sets | Hallmark_Complement | 194 | 1.72 | ＜0.001 | 0.020 |
|  | Hallmark_IL6_Jak_Stat3_Signaling | 87 | 1.81 | ＜0.001 | 0.010 |
|  | Hallmark_Interferon_Alpha_Response | 92 | 1.81 | ＜0.001 | 0.011 |
|  | Hallmark_Mitotic_Spindle | 198 | 1.85 | ＜0.001 | 0.009 |
|  | Hallmark_Allograft_Rejection | 193 | 1.85 | ＜0.001 | 0.010 |
|  | Hallmark_Interferon_Gamma_Response | 194 | 1.86 | ＜0.001 | 0.013 |
|  | Hallmark_E2F_Targets | 190 | 1.76 | 0.004 | 0.017 |
|  | Hallmark_DNA_Repair | 143 | 1.90 | 0.004 | 0.014 |
|  | Hallmark_MYC_Targets_V1 | 184 | 1.97 | 0.008 | 0.016 |
| M1-low Related Gene Sets | None | __ | __ | __ | __ |
| M2-high Related Gene Sets | Hallmark_Epithelial_Mesenchymal_Transition | 195 | 3.10 | ＜0.001 | ＜0.001 |
|  | Hallmark_TGF_Beta_Signaling | 52 | 1.92 | ＜0.001 | ＜0.001 |
|  | Hallmark_Apical_Junction | 187 | 1.97 | ＜0.001 | ＜0.001 |
|  | Hallmark_Kras_Signaling_Up | 192 | 1.95 | ＜0.001 | ＜0.001 |
|  | Hallmark_Protein_Secretion | 95 | 1.75 | ＜0.001 | 0.003 |
|  | Hallmark_Hypoxia | 190 | 1.51 | 0.005 | 0.019 |
|  | Hallmark_Angiogenesis | 35 | 1.67 | 0.008 | 0.004 |
|  | Hallmark_Hedgehog_Signaling | 35 | 1.68 | 0.009 | 0.005 |
| M2-low Related Gene Sets | Hallmark_Mitotic_Spindle | 198 | -1.82 | ＜0.001 | 0.002 |
|  | Hallmark_E2F_Targets | 190 | -3.49 | ＜0.001 | ＜0.001 |
|  | Hallmark_IL6_Jak_Stat3_Signaling | 87 | -1.74 | 0.002 | 0.002 |
|  | Hallmark_DNA_Repair | 143 | -1.75 | ＜0.001 | 0.002 |
|  | Hallmark_G2M_Checkpoint | 185 | -3.12 | ＜0.001 | ＜0.001 |
|  | Hallmark_Myc_Targets_V2 | 55 | -2.51 | ＜0.001 | ＜0.001 |
|  | Hallmark_MTORC1_Signaling | 189 | -2.20 | ＜0.001 | ＜0.001 |
|  | Hallmark_Myc_Targets_V1 | 184 | -2.07 | ＜0.001 | ＜0.001 |
|  | Hallmark_Inflammatory_Response | 198 | -1.55 | ＜0.001 | ＜0.001 |
|  | Hallmark_TNFA_Signaling_Via_NFKB | 194 | -1.71 | ＜0.001 | ＜0.001 |

Supplementary Table 1: The GSEA results of macrophage-related pathways based on microarray data analysis.

Abbreviations: NES=Normalized Enrichment Score, FDR=False Discovery Rate.
